# Contribution of Maternal and Paternal Transmission to Bacterial Colonization in *Nematostella vectensis*

**DOI:** 10.3389/fmicb.2021.726795

**Published:** 2021-10-11

**Authors:** Laura Baldassarre, Shani Levy, Rinat Bar-Shalom, Laura Steindler, Tamar Lotan, Sebastian Fraune

**Affiliations:** ^1^Institute for Zoology and Organismic Interactions, Heinrich-Heine Universität Düsseldorf, Düsseldorf, Germany; ^2^Istituto Nazionale di Oceanografia e di Geofisica Sperimentale - OGS, Sezione di Oceanografia, Trieste, Italy; ^3^Department of Marine Biology, Leon H. Charney School of Marine Sciences, University of Haifa, Haifa, Israel

**Keywords:** vertical transmission, horizontal transmission, marine symbioses, cnidaria, anthozoa

## Abstract

Microbial communities confer multiple beneficial effects to their multicellular hosts. To evaluate the evolutionary and ecological implications of the animal-microbe interactions, it is essential to understand how bacterial colonization is secured and maintained during the transition from one generation to the next. However, the mechanisms of symbiont transmission are poorly studied for many species, especially in marine environments, where the surrounding water constitutes an additional source of microbes. *Nematostella vectensis*, an estuarine cnidarian, has recently emerged as model organism for studies on host-microbes interactions. Here, we use this model organism to study the transmission of bacterial colonizers, evaluating the contribution of parental and environmental transmission to the establishment of bacterial communities of the offspring. We induced spawning in adult male and female polyps of *N. vectensis* and used their gametes for five individual fertilization experiments. While embryos developed into primary polyps, we sampled each developmental stage and its corresponding medium samples. By analyzing the microbial community compositions of all samples through 16S rRNA gene amplicon sequencing, we showed that all host tissues harbor microbiota significantly different from the surrounding medium. Interestingly, oocytes and sperms are associated with distinct bacterial communities, indicating the specific vertical transmission of bacterial colonizers by the gametes. These differences were consistent among all the five families analyzed. By overlapping the identified bacterial ASVs associated with gametes, offspring and parents, we identified specific bacterial ASVs that are well supported candidates for vertical transmission via mothers and fathers. This is the first study investigating bacteria transmission in *N. vectensis*, and among few on marine spawners that do not brood larvae. Our results shed light on the consistent yet distinct maternal and paternal transfer of bacterial symbionts along the different life stages and generations of an aquatic invertebrate.

## Introduction

Multicellular organisms originated in a world dominated by unicellular organisms. Thus, the current-day relationships of animals and microbes, from parasitism to mutualism, evolved most likely from ancient unicellular eukaryote–bacterial interactions (McFall-Ngai et al., 2013; Bosch and McFall-Ngai, 2021). In aquatic environments these relationships are essential components of animal health and physiology, influencing the nutrient cycling (Wegley et al., 2007; Raina et al., 2009; Lema et al., 2012; Santos et al., 2014), gut development (Rawls et al., 2004), resistance against pathogen colonization (Jung et al., 2009; Krediet et al., 2013; Fraune et al., 2015), osmoregulation and oxidative stress responses (Lesser, 1996; Bourne et al., 2016; Peixoto et al., 2017; Rosado et al., 2019), as well as larvae settlement and metamorphosis (Dobretsov and Qian, 2004; Hadfield, 2010; Tran and Hadfield, 2011; Huang et al., 2012).

Given the importance of these relationships, it is essential to understand how bacterial colonization is secured and maintained during the transition from one generation to the next (Bosch and McFall-Ngai, 2021). There are two ways animals acquire their bacterial symbionts, horizontal transmission, in which the bacterial symbionts are acquired from the environment, and vertical transmission in which the bacterial symbionts are transferred via the gametes or by direct contact with the parents. In most animals a combination of both mechanisms (mixed mode transmission) contributes to the establishment of early life bacterial colonization (Bright and Bulgheresi, 2010). While vertical transmission of bacterial symbionts facilitates the evolution and maintenance of mutualistic relationships (Koga et al., 2012; Bosch and McFall-Ngai, 2021), horizontal acquisition requires efficient host selection mechanisms to ensure appropriate bacterial colonization (Nyholm and McFall-Ngai, 2004; Franzenburg et al., 2013). For microbes that are transmitted horizontally, symbiotic life is facultative and free-living populations serve as reservoirs for colonization (Bright and Bulgheresi, 2010). In the marine environment, such free-living populations occur both in shallow and deep waters (Gros et al., 2003; Aida et al., 2008; Harmer et al., 2008) and, in some cases, are replenished by the release of symbionts from the host itself (Salerno et al., 2005). These bacteria provide a diverse pool of potential colonizers for horizontal acquisition and could confer advantages under changing environmental conditions (Hartmann et al., 2017).

As marine invertebrates have great diversity of life history, reproductive and developmental modes, they exhibit diverse modes of bacterial transmission (Russell, 2019). An accredited idea among authors was that species that brood their larvae, transmit symbionts to the next generations through direct contact of the parents with the offspring (Bright and Bulgheresi, 2010; Di Camillo et al., 2012; Bernasconi et al., 2019), while transmission in broadcast spawners, is dominated by horizontal transfer of bacteria from the surrounding water (van Oppen and Blackall, 2019). As more studies are being conducted, there are more evidences that do not necessarily support this idea (Nussbaumer et al., 2006; Apprill et al., 2012; Leite et al., 2017; Björk et al., 2019; Oliveira et al., 2020). Many broadcast spawners pass their symbionts to their offspring by incorporating them into the mucus that envelops oocyte and sperm bundles (Ceh et al., 2012; Ricardo et al., 2016; Leite et al., 2017) and a wide spectrum of mixed-mode transmission in brooders and in free and broadcast spawners is revealed (Sipkema et al., 2015; Fieth et al., 2016; Bernasconi et al., 2019; Damjanovic et al., 2020b).

*Nematostella vectensis* is an anthozoan cnidarian that lives burrowed in sediments of estuarine areas and is widely used as model organism in eco-evo-devo studies (Hand and Uhlinger, 1994; Fraune et al., 2016). *N. vectensis* reproduces both sexually and asexually and its full life cycle can be maintained under laboratory conditions. It is gonochoric and the sexual reproduction is triggered by changes in light exposure, food intake and increased temperature. Adult females release several hundreds of oocytes embedded in gelatinous sacks, while adult males release sperms directly into the surrounding water (Hand and Uhlinger, 1992; Fritzenwanker and Technau, 2002; Stefanik et al., 2013). The embryos develop inside the gelatinous sack and, within 1 to 2 days, ciliated planula larvae hatch from the oocytes and is released into the water where they remain freely swimming until settlement. After settlement, the planulae metamorphose into primary polyps. Under optimal conditions, the sexual maturity is reached within 3–4 months (Hand and Uhlinger, 1992).

Previous studies showed that adult *N. vectensis* harbors a specific microbiota whose composition changes in response to different environmental conditions and among geographic locations (Mortzfeld et al., 2016). In addition, sampling of different body regions (physa, mesenteries and capitulum) of the adult revealed a specific microbiota for each region, with specific dominance of spirochaetes bacteria within the capitulum (Bonacolta et al., 2021). Also changes in the diet lighting cycle induced differences in composition and relative abundance in *N. vectensis* microbiome (Leach et al., 2019). It has also been observed that different life stages (larva, juvenile, and adult) host specific microbiota (Mortzfeld et al., 2016; Domin et al., 2018).

In this study, we aimed at understanding how microbial symbionts are transmitted through generations and established in early life stages. Through metabarcoding of the 16S rRNA gene, we analyzed the microbial community composition in separated pairs of adult female and male polyps, and their corresponding newly released gametes, planula larvae and primary polyps. The comparisons to the corresponding medium microbiota allowed us to identify bacterial species that are specifically host associated and putatively maternally and paternally transmitted to the offspring.

## Materials and Methods

### Animal Culture

*Nematostella vectensis* anemones were F1 offspring of CH2 × CH6 individuals collected from the Rhode River in Maryland, United States (Hand and Uhlinger, 1992; Fritzenwanker and Technau, 2002). Adult polyps were cultured in *N. vectensis* medium (NM) composed of 12.5 ppt artificial sea water (Red Sea) and maintained at 18°C in the dark. They were fed five times a week with freshly hatched *Artemia* brine shrimps (Ocean Nutrition Sep-Art Artemia Cysts). Embryos were raised at 21°C in the dark and planulae or polyps were collected.

### Spawning Induction and Fertilization

**FIGURE 1:**
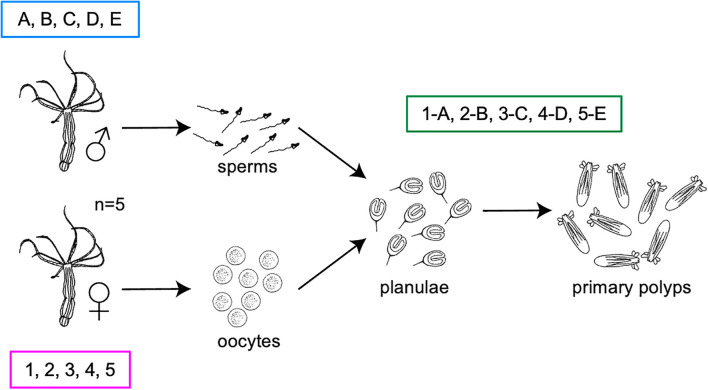
Experimental design. Five pairs of parent polyps were induced for spawning. Their gametes and offspring were collected separately and used for further development and 16S rRNA gene sequencing.

Five adult female polyps (labeled with numbers) and five adult male polyps (labeled with letters) were starved for 4 days prior to fertilization at standard conditions (18°C, in the dark), to avoid contamination from the food. A day before induction the animals were washed three times with sterile NM (autoclaved and filtered on 0.22 μm membrane) and separated into sterile six well plates. The induction was performed by exposing both males and females to increased temperature (25°C) and light for 13 h. After spawning, the adults were washed three times with sterile NM, snap-frozen in liquid nitrogen and stored at −80°C until DNA extraction. As control, the media where each female was kept before and during induction was filtered on a 0.22 μm membrane and stored at −80°C for DNA extraction. Sperms and oocyte sacks were individually collected into 1.5 ml tubes. Half of the sperms from each male polyp was collected for DNA extraction and the other half was used for fertilization of the oocytes. The sperms for DNA extraction were washed three times in sterile NM and stored at −80°C until processing. Each oocyte sack (here referred as oocytes) was washed three times in sterile NM and cut in two halves using a sterile scalpel; one half was collected for DNA extraction and the other half was fertilized. Four days post-fertilization (dpf), fertilized oocytes developed into planulae. Half of the planulae from each oocyte sack was washed three times with sterile NM and collected for DNA extraction, the other half was kept in the incubator for further development. Ten dpf, planulae developed into primary polyps that were washed three times with NM and collected for DNA extraction (Figure 1).

### DNA Extraction and 16S rRNA Sequencing

The gDNA was extracted from adult animals, oocyte sacks, sperms, planulae, and primary polyps, with the DNeasy^®^ Blood and Tissue Kit (Qiagen, Hilden, Germany) as described in the manufacturer’s protocol. DNA was eluted in 100 μL elution buffer. The eluate was kept frozen at −20°C until sequencing. For each sample the hypervariable regions V1 and V2 of bacterial 16S rRNA genes were amplified. The forward primer (5′- **AATGATACGGCGACCACCGAGATCTACAC** XXXXXXXX TATGGTAATTGT AGAGTTTGATCCTGGCTCAG-3′) and reverse primer (5′- **CAAGCAGAAGACGGCATACGAGAT** XXXXXXXX AGTCAGTCAGCC TGCTGCCTCCCGTAGGA GT -3′) contained the Illumina Adaptor (in bold) p5 (forward) and p7 (reverse) (Fadrosh et al., 2014). Both primers contain a unique 8 base index (index; designated as XXXXXXXX) to tag each PCR product. For the PCR, 100 ng of template DNA (measured with Qubit) were added to 25 μl PCR reactions, which were performed using Phusion^®^ Hot Start II DNA Polymerase (Finnzymes, Espoo, Finland). All dilutions were carried out using certified DNA-free PCR water (JT Baker). PCRs were conducted with the following cycling conditions [98°C—30 s, 30 × (98°C—9 s, 55°C—60 s, 72°C—90 s), 72°C—10 min] and checked on a 1.5% agarose gel. The concentration of the amplicons was estimated using a Gel Doc TM XR + System coupled with Image Lab TM Software (BioRad, Hercules, CA, United States) with 3 μl of O’GeneRulerTM 100 bp Plus DNA Ladder (Thermo Fisher Scientific, Inc., Waltham, MA, United States) as the internal standard for band intensity measurement. The samples of individual gels were pooled into approximately equimolar sub-pools as indicated by band intensity and measured with the Qubit dsDNA br Assay Kit (Life Technologies GmbH, Darmstadt, Germany). Sub-pools were mixed in an equimolar fashion and stored at −20°C until sequencing. Sequencing was performed on the Illumina MiSeq platform with v3 chemistry (Rausch et al., 2016). The raw data are deposited at the Sequence Read Archive (SRA) and available under the project ID PRJNA737505.

### Analyses of Bacterial Communities

A total of 35 samples belonging to five separated families (five mothers, five fathers, five sperm batches, five oocyte sacks, five planulae batches, five primary polyps batches, and five medium controls) were submitted for 16S RNA gene sequencing. The 16S rRNA gene amplicon sequence analysis was conducted through the QIIME2 2021.4 platform (Bolyen et al., 2019). Sequences with 100% identity were grouped into ASVs and clustered against the SILVA RNA reference database (Quast et al., 2013; Yilmaz et al., 2014). Denoising and quality filtering were performed through the DADA2 plugin implemented in QIIME2 (Callahan et al., 2016, p. 2). A sample with less than 7000 reads was removed from the dataset, being considered as outlier. For the successive analysis, the number of ASVs per sample was normalized to 7000 reads.

Alpha-diversity was calculated using the Faith’s PD, evenness, dominance and the total number of observed ASVs metrics implemented in QIIME2. Statistical significance was tested through the non-parametric Kruskal-Wallis test through QIIME2 and JASP 0.14.1 (JASP Team, 2020).

Beta-diversity was calculated in QIIME1 (Caporaso et al., 2010) and QIIME2 according with the different β-diversity metrics available (Binary-Pearson, Bray-Curtis, Jaccard, Weighted-Unifrac, and Unweighted-Unifrac). Statistical values of clustering were calculated using the non-parametric comparing categories methods PERMANOVA and Anosim implemented in QIIME2.

Bacterial ASVs associated with specific conditions were identified through LEfSe (Segata et al., 2011). LEfSe uses the non-parametric factorial Kruskal-Wallis sum-rank test to detect features with significant differential abundance, with respect to the biological conditions of interest; subsequently LEfSe uses Linear Discriminant Analysis (LDA) to estimate the effect size of each differentially abundant feature. To identify vertically transmitted bacteria ASVs, we performed pairwise comparisons of the surrounding medium microbiota with the microbiota of the *N. vectensis* samples. We were then able to infer ASVs associated with each animal life stage and therefore putative bacterial candidates for vertical transmission. The results obtained from LEfSe analyses were cross-checked against the outcomes of a logical test based on presence/absence data, performed directly on the ASV table generated for each sampled family. We assumed that a maternally transmitted bacterium should be present simultaneously in the microbiota of mother polyps, oocytes and planulae but absent from the medium and a paternally transmitted bacterium should be present in father polyps, sperms and planulae but absent from the medium. Bacterial ASVs that might be acquired horizontally from the medium by the early life stages were filtered by their concurrent presence in planula or primary polyps and medium and absence in the gametes.

## Results

### Microbiota Associated With *N. vectensis* Tissues Is Distinct From Surrounding Medium

The experimental setup allowed us to assign gametes and offspring to their parent polyps in order to identify bacterial colonizers that are likely transmitted vertically among separated animal families and to differentiate them from the surrounding medium (Figure 1).

**FIGURE 2:**
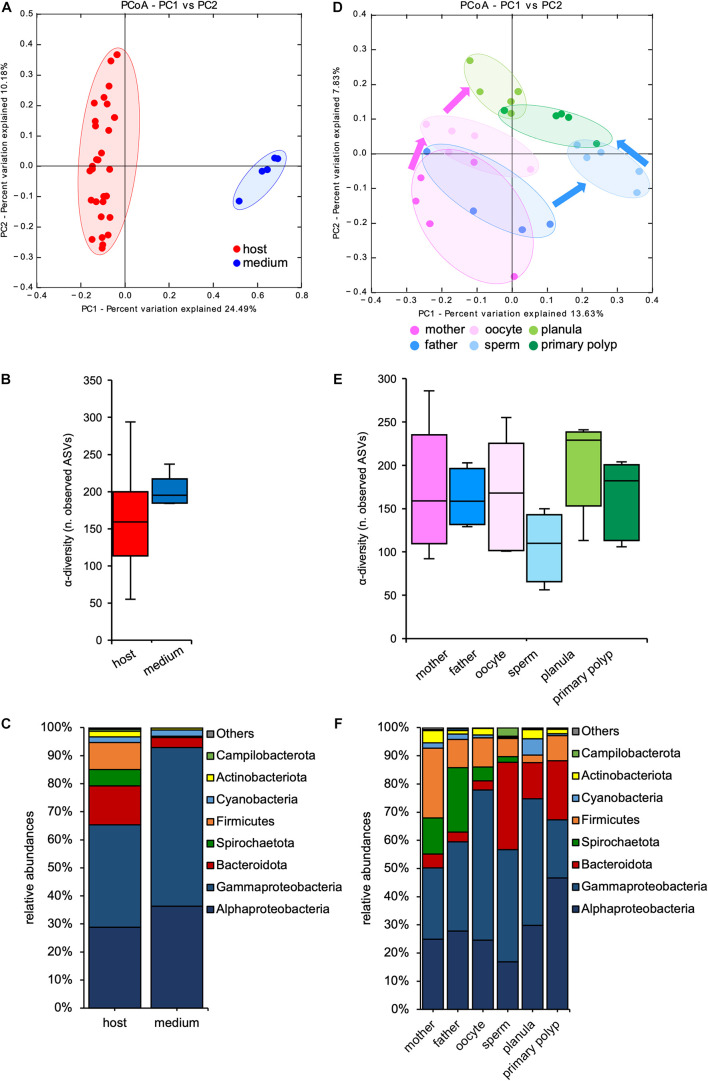
Microbiota diversity analyses among sample source and life stage. **(A)** PCoA (based on Binary-Pearson metric, sampling depth = 7000) illustrating similarity of bacterial communities based on sample source; **(B)** α-diversity (observed ASVs) comparison of medium and animal tissue samples (max rarefaction depth = 7000, num. steps = 10); **(C)** relative abundance of main bacterial groups between host and medium samples; **(D)** PCoA (based on Binary-Pearson metric, sampling depth = 7000) illustrating similarity of bacterial communities based on developmental stage; **(E)** α-diversity (observed ASVs) comparison by developmental stage (max rarefaction depth = 7000, num. steps = 10); **(F)** relative abundance of main bacterial groups among different life stages.

The sequencing was successful for 34 samples. After filtering and denoising, 2325 different ASVs were detected, with the number of reads per sample ranging between 678748 and 7026 (Supplementary Table 1). Beta-diversity analyses revealed that bacterial communities from the surrounding medium were distinct from those associated with host tissue (Figure 2A and Table 1), indicating a specific bacterial colonization in all life stages of *N. vectensis*. In contrast, α-diversity analyses revealed no significant differences between the bacterial communities of the medium to the bacterial communities associated with host tissues (Figure 2B and Supplementary Table 2). Although the medium showed a similar species richness compared to the animal tissues (Figure 2B), bacterial species in the medium showed lower diversity at the phylum level compared to those associated with the host (Figure 2C), e.g., Spirochaetota and Firmicutes are absent in the medium. In comparison, the host bacterial communities showed a higher evenness and lower dominance (Figure 2C and Supplementary Table 2) and harbored uniquely more bacterial species than the medium, with an overlap of 161 ASVs shared between both medium and host (Supplementary Figure 1). Given that the life stages of *N. vectensis* associate with specific bacterial communities distinct from the environment, a portion of these is likely to be non-random.

**TABLE 1:** Beta-diversity statistical tests comparing the different sample sources, the developmental stages and the families.

		ANOSIM		PERMANOVA	
Parameter	β-diversity metric	*R*	*p-value*	*pseudo-F*	*p-value*
host vs. medium	Binary-Pearson	0.987	0.001	10.130	0.001
	Bray-Curtis	0.940	0.001	9.757	0.001
	Jaccard	0.978	0.001	6.048	0.001
	Weighted-Unifrac	0.198	0.069	5.800	0.001
	Unweighted-Unifrac	0.865	0.001	6.559	0.001
life stage	Binary-Pearson	0.408	0.001	1.926	0.001
	Bray-Curtis	0.488	0.001	2.682	0.001
	Jaccard	0.458	0.001	1.544	0.001
	Weighted-Unifrac	0.468	0.001	3.553	0.001
	Unweighted-Unifrac	0.339	0.001	1.614	0.001
Family	Binary-Pearson	0.004	0.420	1.041	0.347
	Bray-Curtis	0.000	0.465	0.984	0.470
	Jaccard	−0.030	0.682	1.020	0.353
	Weighted-Unifrac	−0.031	0.698	0.978	0.467
	Unweighted-Unifrac	−0.031	0.683	0.975	0.573

*Statistical *analyses* were performed (methods ANOSIM and PERMANOVA, number of permutations = 999) on each of the pairwise comparison distance matrices generated.*

### Specific Bacterial Communities Colonize Oocytes and Sperms

Analyzing the associated bacterial communities of *N. vectensis* according with their life stages revealed a clear clustering (Figure 2D and Table 1). Interestingly, the bacterial communities of sperms and oocytes were distinct from those of all the other life stages and from each other, indicating distinct mechanisms shaping the bacterial colonization of gametes (ANOSIM: *R* = 0.444, *p* = 0.003, Figure 2D), and a specific transmission from male and female parent polyps. Both planulae and primary polyps harbored similar bacterial communities (Figure 2D) that clustered between the sperms and oocytes samples, suggesting the contribution of maternal and paternal transmitted bacteria to early life stages colonization. While no significant differences in the bacterial α-diversity could be detected, sperms harbored bacterial communities with a slightly lower bacterial α-diversity compared to all the other samples (Figure 2E and Supplementary Table 3).

In contrast, no clustering (Supplementary Figure 2A and Table 1) and no differences in α-diversity (Supplementary Figure 2B and Supplementary Table 4) were evident according to family status.

Looking at the different bacterial groups, greater abundances of Spirochaetota (between 23.2 ± 17.2% and 5.2 ± 5.9%) and Firmicutes (between 27.3 ± 13.6% and 10.4 ± 7.8%) were evident in the adults and in the oocytes, while Bacteroidota were relatively more abundant in the sperms and in the offspring (between 27.6 ± 29.2% and 12.4 ± 12.4%). The abundance of Alphaproteobacteria increased from the adults (23.4 ± 10.9% and 27.2 ± 13.9%, respectively) through the primary polyps, in which they reached the maximum abundance (46.4 ± 9.3%) (Figure 2F). These differences among life stages and sexes suggest a differential transmission of specific bacterial groups through the gametes.

### Offspring Bacterial Colonizers Originate From Oocytes, Sperms, and Surrounding Medium

**FIGURE 3:**
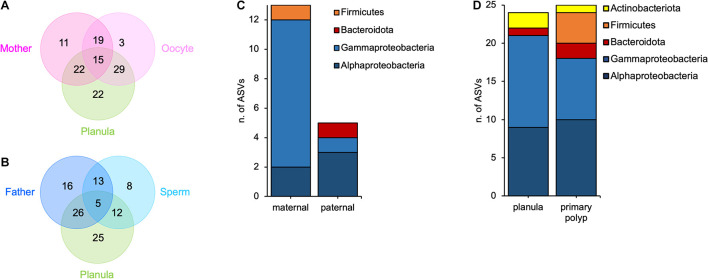
Vertical and horizontal contributions to offspring microbiota. **(A)** Venn diagram showing the number of ASVs shared between mothers, oocytes and planulae; **(B)** Venn diagram showing the number of ASVs shared between fathers, sperms and planulae; **(C)** Bar-chart of parentally transmitted bacterial ASVs divided by major groups; **(D)** Bar-chart of environmentally transmitted bacterial ASVs divided by major groups.

By using the LEfSe algorithm (Segata et al., 2011) and the pairwise comparisons between host tissues and surrounding medium, we identified 15 ASVs that were significantly more abundant in mother polyps, oocytes, and planulae (Figure 3A and Supplementary Table 5) and 5 ASVs that were significantly more abundant in father polyps, sperms and planulae (Figure 3B and Supplementary Table 5), than in the surrounding medium. By overlapping these results with those obtained from the presence-absence calculations (Supplementary Table 1), we were further able to filter the LEfSe results to those candidates that were completely absent from the medium in any of the animal families (Supplementary Table 6). We ended up with 13 ASVs potentially transmitted by the mother and 5 ASVs potentially transmitted by the father (Figure 3C). The ASVs potentially transmitted by both mother and father polyps belong to the classes Alpha- and Gammaproteobacteria (Supplementary Table 6). In addition, one ASV potentially transmitted by mother polyps is a member of the Firmicutes phylum, while father polyps potentially transmit one member of the Bacteroidota phylum (Figure 3C). The high resolution of the presence-absence analysis, allowed us to also point out a slight variability in bacterial transmission that occur between different families (Supplementary Table 6).

In a final step we aimed at detecting bacterial ASVs that are present in the offspring and in the medium but not in the gametes, and thus, likely acquired horizontally from the medium. For planulae and primary polyps, we detected 24 and 25 ASVs, respectively, with an overlap of 10 ASVs shared between both developmental stages (Supplementary Table 6). In both planulae and primary polyps, these bacteria belonged to the classes Alpha- and Gammaproteobacteria, and the phyla Bacteroidota and Actinobacteriota. In addition, the primary polyps shared with the medium also four members of the Firmicutes (Figure 3D). These results suggest that each developmental stage is able to associate with different and specific environmental symbionts.

## Discussion

### Maternal and Paternal Transmission of Bacterial Symbionts

A reliable transfer of specific symbionts is required to maintain the bacterial associations across generations. Thereby, vertically transmitted bacteria may represent beneficial symbionts, necessary for animals’ development and physiology, bacteria that lack a free-living stage, or more simply, bacteria that are present in the open water in too low abundance to be recruited (Salerno et al., 2005; Bright and Bulgheresi, 2010). So far, few studies have undertaken a detailed comparison of microbial communities of parents, gametes and progeny in marine organisms (Sipe et al., 2000; Bright and Bulgheresi, 2010; Sharp et al., 2012; Leite et al., 2017; Quigley et al., 2017, 2018, 2019; Bernasconi et al., 2019; Damjanovic et al., 2020a; Oliveira et al., 2020; Igawa-Ueda et al., 2021).

Our results, consistent with results obtained in former studies on broadcast spawning corals (Sharp et al., 2010; Quigley et al., 2017), indicate that *N. vectensis* transmits microbial symbionts to its offspring mainly maternally. Current knowledge is limited on the contribution of male parents to the progeny microbiota (Damiani et al., 2008; Watanabe et al., 2014; De Vooght et al., 2015); nevertheless, some examples exist regarding marine invertebrates (Usher et al., 2005; Sharp et al., 2010; Padilla-Gamiño et al., 2012; Kirk et al., 2013; Leite et al., 2017). In our study it seems that also male polyps transmit specific bacteria to the next generation.

As suggested from previous studies on corals, we hypothesize that in *N. vectensis* mother polyps incorporate bacterial colonizers into the mucus bundles that surround the oocytes (Ceh et al., 2012; Ricardo et al., 2016; Leite et al., 2017), while the sperms may acquire the bacteria before fertilization by horizontal transmission through water, which contains bacteria released by the parents (van Oppen and Blackall, 2019). This strategy is less specific than strict vertical transfer, however, not as non-specific as random horizontal acquisition of seawater communities (Ceh et al., 2013).

Our results are supported by fluorescence *in situ* hybridization (FISH) approaches applied on coral larvae and gametes, indicating the presence of bacteria in the ectoderm of brooded larvae (Sharp et al., 2012), but not inside gametes, embryos, and larvae of several broadcast spawners (Sharp et al., 2010).

### Offspring Microbiota Results From Mixed-Mode Bacterial Transmission

*Nematostella vectensis* male and female polyps transmit different bacterial species through their gametes, with the oocytes contributing the most to the bacterial assemblage of the planulae, thus indicating distinct selecting forces. For instance, through the oocyte bundles, the mothers might provide the developing embryos with specific antimicrobial peptides (Fraune et al., 2010) and, in the case of *N. vectensis* also with nematosomes, multicellular motile bodies with putative defense function (Babonis et al., 2016). In vertebrates, like birds, fishes, and reptiles, passive immunity is transmitted through the deposition of antibodies in eggs (Grindstaff et al., 2003) and the fertilization envelope of fish eggs has demonstrated bactericidal activity (Kudo and Inoue, 1989).

Although not yet demonstrated, the bigger size of oocytes and the presence of a cytoplasm, in comparison to the sperm, may offer more room for carrying symbionts on the surface and/or intracellularly; sperms on the other hand, may carry strict symbionts in the nucleus (Usher et al., 2005; Watanabe et al., 2014). Additionally, the offspring can be partly colonized post-spawning through the uptake of microbial associates released by the parents into the surrounding seawater, as previously described (Apprill et al., 2009; Sharp et al., 2010; Ceh et al., 2012, 2013).

As already observed, the early life stages and adult microbiota in *N. vectensis* differ significantly (Mortzfeld et al., 2016; Domin et al., 2018). Our results suggest that a portion of the early life stages microbiota is the result of a parental transmission while another part is horizontally acquired from the environment during ontogeny. This hypothesis is supported also by the tendency of higher bacterial species richness associated with the early developmental stages in comparison to that of the adults. Higher α-diversity in the early life stages has been described also in other studies on marine animals (Nyholm and McFall-Ngai, 2004; Littman et al., 2009; Fieth et al., 2016; Mortzfeld et al., 2016; Epstein et al., 2019) indicating an host filtering window during which the microbiota is shaped to a more stable community. This can relate with the different ecological (e.g., pelagic vs. benthonic, motile vs. sessile, preying vs. filter-feeding) and/or developmental requirements across the life stages of animals that have complex life cycles (Mortzfeld et al., 2016; Bosch and McFall-Ngai, 2021; Putnam, 2021). *N. vectensis’* life cycle includes a pelagic, freely swimming, not feeding planula larva and benthonic, sessile, preying primary polyp and adult stages. Therefore, it is easy to imagine that the symbiotic microbial community is impacted by the deep ecological and morphological changes during host development and that specific bacterial species may provide different benefits to the different life stages. As already pointed out (Fraune et al., 2010), organisms in which embryos develop outside the mother’s body, in a potentially hostile environment, use a “be prepared” strategy involving species-specific, maternally produced antimicrobial peptides for protection. These antimicrobial peptides not only have bactericidal activity but also actively shape and select the colonizing bacterial community (Fraune et al., 2010; Fieth et al., 2016). It is likely that also *N. vectensis* is able to employ different mechanisms to shape and control its symbiotic microbiota in a life stage-specific manner.

Consistently with previous studies (Gilbert et al., 2012; Deines et al., 2017; Hernandez-Agreda et al., 2017; Goldsmith et al., 2018; Sullam et al., 2018; Glasl et al., 2019; Berg et al., 2020), our results showed that between different parents a slight variability of vertically and environmentally transmitted bacteria exists, highlighting the potential impact of host genotype and stochastic events on symbiotic community establishment of offspring.

The hologenome theory of evolution (Zilber-Rosenberg and Rosenberg, 2008) proposed that microbiome mediated plasticity of the host phenotype can be under selection pressure and may contribute to animal adaptation. Vertical transmission of the microbiota could therefore facilitate transgenerational adaptation of animals to changing conditions (Webster and Reusch, 2017), while horizontal transmission may mitigate some of the deleterious consequences of obligate host-association/strict vertical transmission (e.g., genome degradation and reduction of effective population size) (Russell, 2019). Concertedly, vertical transmission may secure the maintenance of coevolved beneficial bacteria, while horizontal acquirement of new bacterial partners increases the flexibility of beneficial effects under changing environmental conditions. Future studies should compare the function of vertically transmitted and horizontally acquired bacterial associates, providing important insights into the potential of microbial communities to promote animal adaptation to changing environmental conditions.

## Data Availability Statement

The datasets presented in this study can be found in online repositories. The names of the repository/repositories and accession number(s) can be found in the article/Supplementary Material.

## Author Contributions

All authors contributed to conception and design of the study, manuscript revision, read, and approved the submitted version. LB, SL, and RB-S performed the experiment. LB performed the statistical analysis. LB and SF wrote the first draft of the manuscript.

## Conflict of Interest

The authors declare that the research was conducted in the absence of any commercial or financial relationships that could be construed as a potential conflict of interest.

## Publisher’s Note

All claims expressed in this article are solely those of the authors and do not necessarily represent those of their affiliated organizations, or those of the publisher, the editors and the reviewers. Any product that may be evaluated in this article, or claim that may be made by its manufacturer, is not guaranteed or endorsed by the publisher.
