# Supplementary material for: Contribution of Maternal and Paternal Transmission to Bacterial Colonization in Nematostella vectensis
Source: Front Microbiol. 2021 Oct 11;12:726795. doi: 10.3389/fmicb.2021.726795 (PMC8544946; doi:10.3389/fmicb.2021.726795)
Supplement: Supplementary file 1 [file Data_Sheet_1.PDF]

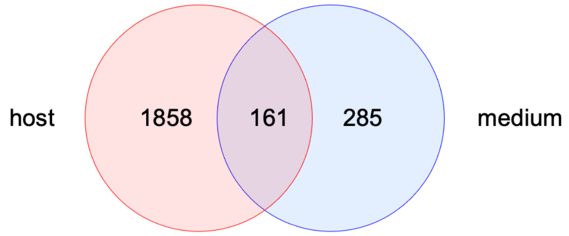

Figure S1. Number of ASVs specific for the host samples, the surrounding medium and those shared between both.

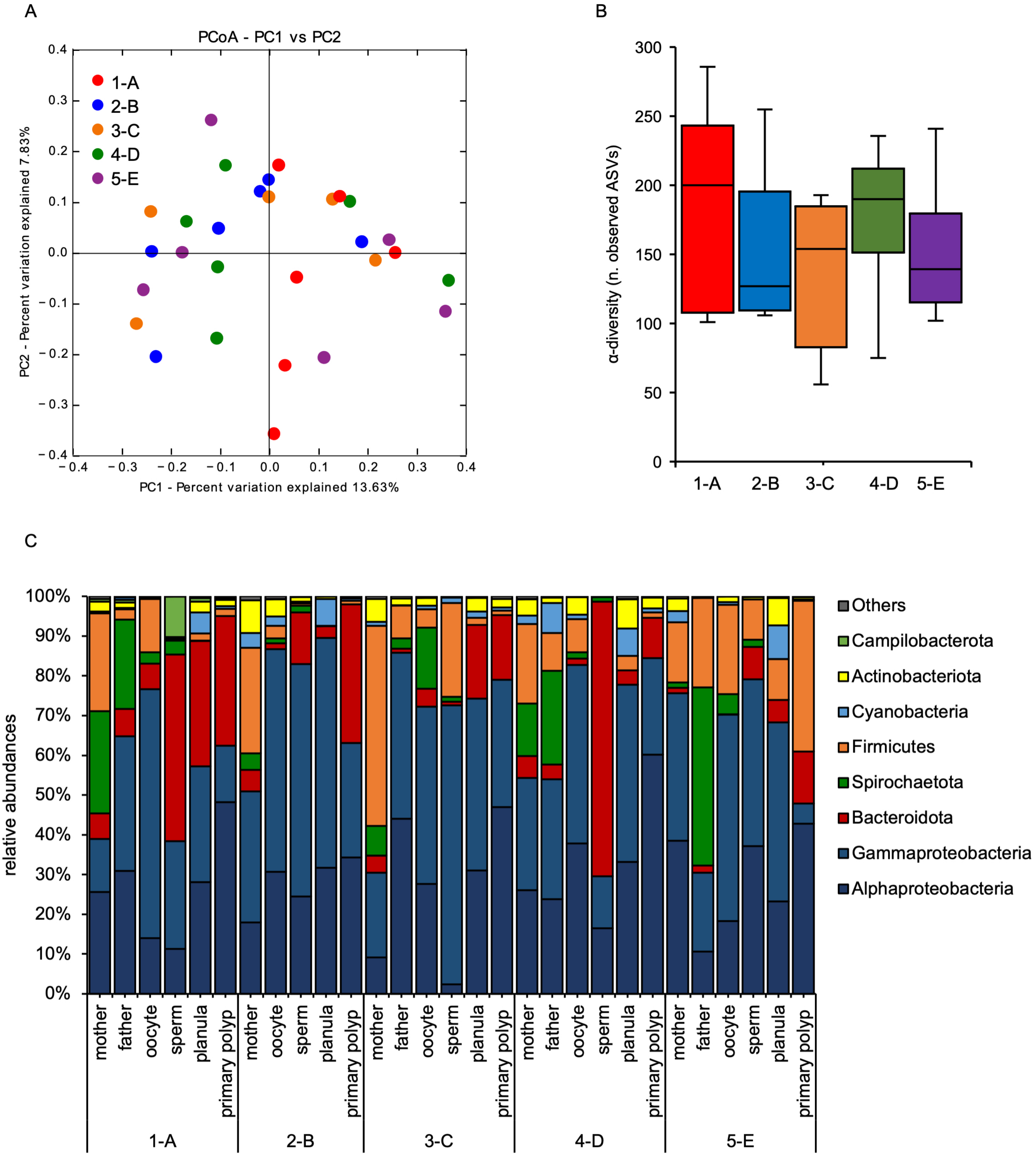

Figure S2. Microbiota diversity analyses among animal family. (A) PCoA (based on Binary-Pearson metric, sampling depth = 7000) illustrating similarity of bacterial communities based on animal family; (B) α-diversity (observed ASVs) comparison between sample animal families (max rarefaction depth = 7000, num. steps = 10); (C) major bacterial groups divided by family and developmental stage.
